# Use of Plant Protection Products in Lombardy, Italy and the Health Risk for the Ingestion of Contaminated Water

**DOI:** 10.3390/toxics9070160

**Published:** 2021-07-06

**Authors:** Rosa Mercadante, Beatrice Dezza, Teresa Mammone, Angelo Moretto, Silvia Fustinoni

**Affiliations:** 1 EPIGET—Epidemiology, Epigenetics, and Toxicology Laboratory, Department of Clinical Sciences and Community Health, Università degli Studi di Milano, 20122 Milano, Italy; rosa.mercadante@unimi.it (R.M.); beatrice.dezza@gmail.com (B.D.); 2 International Centre for Pesticides and Health Risk Prevention (ICPS) ASST Fatebenefratelli Sacco, 20157 Milano, Italy; mammone.teresa@hsacco.it (T.M.); angelo.moretto@unipd.it (A.M.); 3 Department of Biomedical and Clinical Sciences, Università degli Studi di Milano, 20157 Milano, Italy; 4 Department of Cardio-Thoraco-Vascular and Public Health Sciences, Università degli Studi di Padova, 35128 Padova, Italy; 5 Environmental and Industrial Toxicology Unit, Fondazione IRCCS Ca’ Granda Ospedale Maggiore Policlinico, 20122 Milano, Italy

**Keywords:** plant protection products, pesticides exposure, groundwater contamination, human health risk assessment, ingestion of contaminated water

## Abstract

Pesticides used to protect agricultural crops may contaminate groundwater. This work aimed to identify the pesticides used in Lombardy, Italy, in 2016, their concentration in the groundwater and the risk for health associated with the intake of drinkable water in the adult population. The risk was evaluated for the presence of single and multiple active substances in the groundwater, calculating the hazard quotient (HQ) and the hazard index (HI), respectively. Lombardy utilises an agricultural area of 980,112 h, which is mainly cultivated with cereals (74%). Approximately 2354 pesticides (about 1.3 × 10^7^ kg), containing 410 active substances (about 4.5 × 10^6^ kg) were sold. There were groundwater contamination measurements in 158 monitoring points, which were investigated twice a year for 31 active substances, and a total of 9152 determinations. Only 17 currently used active substance were measured in the groundwater, among which three belonged to the 10 best-sold pesticides. The exceedance of the environmental quality standard was observed for about 1.5% determinations. The intake of contaminated water in the adult population resulted in a HQ typically ranging between 10^−3^ and 10^−4^ and a HI of about 10^−3^. Although the number of pesticides sold in 2016 in Lombardy was big, only a small fraction of active substances was monitored in the groundwater. Considering these monitored substances, the intake of contaminated groundwater in the adult general population posed an irrelevant risk for health.

## 1. Introduction

Plant protection products (PPPs) are mixtures of substances widely used worldwide and voluntarily spread in the environment for the protection of crops from harmful agents. PPPs contain one or more active substances: the molecules that are responsible for the toxic action towards pests. In Italy, about 150,000 tons/year of PPPs, corresponding to about 60,000 tons/year of active substances, are used [1].

Active substances are subject to authorisation, in accordance with the European legislation [2,3,4], which aims to ensure a safe use of pesticides for both humans and the environment. Despite this, the great use of these chemicals, their widespread presence in the environment, and toxicity raise concern for human health.

Lombardy is one of the regions of Italy with the largest agricultural areas, including the Po Valley (about 47% of the regional area), mountains (about 41%), and a hilly area (12%). The crops present in the Po Valley are mainly cereals, while in the hilly area, the most common crop is vineyard. The farming is predominantly traditional; organic farming has been introduced only in recent years, and it is practiced on about 5% of the territory [5].

In Lombardy, a registry of sales of PPPs was established in 2015 at the International Centre for Pesticides and Health Risk Prevention (ICPS) [6]; this registry is fed by the information provided every year by the local stores, as required by the regional law (DPR 290/01, art. 42) [7].

One aspect connected with the use of PPPs is the protection of water bodies. The European Union Directive 2006/118/EC [8], in Italy implemented with the D.Lgs. 30/2009 [9], establishes a regime that sets environmental quality standards (EQS) for groundwater. The EQS for pesticide active substances are 0.1 µg/L (EQS_single_) and 0.5 µg/L (EQS_multiple_) for a single and for the sum of two or more active substances, respectively.

The monitoring network of the Environmental Regional Protection Agency [10] controls all water bodies that flow into the subsoil of the region through a quantitative monitoring to verify that resources are not running out and to ensure that water is not polluted. At the time of this study, the criteria for the identification of PPPs to be submitted to control were given by the guideline of the Italian National System for the Environmental Protection [11], dealing with the design of networks and water monitoring programs according to the national law D.Leg. 152/2006 [12]. In 2018, a new refined guideline, specifically devoted to PPPs, was emitted [13]. In this document, the criteria for the identification of PPPs are based on the list of chemicals included in the EU regulation [14], the use of PPPs in the territory, their previous occurrence in the groundwater according to the monitoring campaigns, the affinity for the water, and the classification of hazard according to the Regulation (EC) No 1272/2008—classification, labeling, and packaging of substances and mixtures (CLP) [15].

In order to assess the risk for human health associated with the use of pesticides, the estimate of the dietary daily intake is compared with the Acceptable Daily Intake (ADI). The ADI is the dose of an active substance, expressed in mg per kg body weight, which can be taken daily for the whole life without adverse effects. ADIs are set, internationally, by the Joint FAO/WHO Meeting on Pesticide Residue [16] or by national or supranational bodies (e.g., the European Union). As a convention, for the intake via drinking water, 20% of the ADI is allocated [17].

Within the Lombardy region, this study aimed to evaluate the type and quantity of PPPs sold in 2016 and the active substances there contained. Moreover, the study retrieved the concentrations of active substances in the groundwater and assessed the health risk associated with the intake of contaminated water in the adult general population.

## 2. Materials and Methods

### 2.1. Lombardy Agricultural Territory

The information on the total area of the Lombardy territory was obtained from the Lombardy Regional Statistical Yearbook, which was updated in November 2017 [18]. The information on the total agricultural area (SAT), the agricultural used area (SAU), and the area dedicated to the different crops was obtained from the databases Wharehouse of the 6th Census of Italian Agriculture of the Italian Institute of Statistics [19]. The considered crop classes were cereals (identified in the original dataset as “seminative”), fruit and vegetables, and vine and grass (grassland, pastures).

### 2.2. Plant Protection Products: Sale Data and Toxicity

The commercial names and quantities of PPPs sold in Lombardy in 2016 were obtained by the fitoWeb290 website of the International Centre for Pesticides and Health Risk Prevention of Lombardy [6]. From the composition of PPPs, as indicated on the label, the names of the active substances and their quantities, broken down by functional class (herbicide, fungicide, insecticide, multi action, mix), and the target crops, were derived. PPPs were also distinguished into organic (i.e., allowed for organic farming) and non-organic (i.e., registered for conventional farming) through the consultation of the database of organic products [20]. In order to assess the impact of these substances on the territory, the quantities of PPPs sold in Lombardy and the agricultural used area were used to calculate the density of use (kg/h).

For the ten best-sold non-organic active substances, information on their hazard classification, which was expressed through hazard statements (Table S1), according to the European Community Regulation no. 1272/2008, were retrieved [21].

### 2.3. Plant Protection Products in Groundwater

In order to identify the active substances that are actually sought in groundwater by official controls, ARPA Lombardia’s data on the monitoring of quality of groundwater bodies were scrutinised. ARPA Lombardia’s groundwater monitoring network consists of 496 monitoring points for the qualitative analysis, covering the whole region and all the underground water bodies. Of these, pesticides are measured in a subset of 158 monitoring points, which were all included in the present study. Given the great variety of situations in which water samples are taken, there is not just a single withdrawal approach, but there are different procedures described in a general protocol [22]. The results of controls carried out in the year 2016, for in the spring (from April to July) and autumn (from September to December) campaigns, were consulted [10]. The names of the active substances and/or their metabolites (monitored molecules) and their concentrations were extracted.

The number of monitoring points where the EQS for a single molecule was exceeded [8] was calculated (EQS_single_ = 0.1 µg/L). The monitored molecules with at least one exceedance were identified, the number of exceedances was calculated, and their mean and maximum concentrations were recorded. Moreover, the monitoring points with two or more detected monitored molecules were identified. The monitoring points where the sum of the concentrations of active ingredients exceeded the EQS for multiple molecule (EQS_multiple_ = 0.5 µg/L) were recorded [8]. For each active ingredient and/or metabolite exceeding the EQS, the hazard statement was retrieved [15].

### 2.4. Health Risk Assessment

According to the World Health Organisation guidelines for water intended for human use, 20% of the maximum acceptable daily intake (ADI) was considered, which indicates that drinking water contributes one-fifth of the total daily intake [16]. For the single active substances and/or their metabolites with at least one exceedance of the EQS_single_, the risk was estimated as Hazard Quotient (HQ) using the following formula:

(1) $$HQ=\frac{C\;\left(\frac{µg}{L}\right)\times 2\;L}{70\;\mathrm{kg}\times 0.2\times ADI\;\left(\frac{\mathrm{mg}}{\mathrm{kg}}\right)\;}\times 10^{-3}$$

where *C* is the concentration of the active ingredient and/or metabolite in the groundwater, 70 kg is the average weight of an adult individual, and 2 L is the mean volume of water ingested per day. The risk is considered acceptable when *HQ* < 1. This estimate has been performed using both the mean and the maximum concentration of active ingredient and/or metabolite in the groundwater, obtaining HQ_mean_ and HQ_max,_ respectively.

For monitoring points where the sum of two or more active substances and/or their metabolites exceeded the EQS_multiple_, the risk was estimated following a previously proposed approach [23]. Active ingredients and/or their metabolites were grouped based on similar adverse end points/critical effects. Specifically, molecules with systemic toxicity (hazard statements H351 and H373) and the molecules with local toxicity (hazard statement H317) were grouped [15]. The risk of the mixture was estimated as Hazard Index (HI), using the following formula:

(2) $$HI=\overset{n}{\underset{i=2}{\sum }}HQi$$

where *n* is the number of active ingredients and/or metabolites with similar end points/critical effects. The *HI* was evaluated separately for local or systemic toxicity.

## 3. Results

### 3.1. Lombardy Agricultural Territory

Table 1 shows the results of the analysis of the territory of Lombardy, in which there are 12 provinces and 1539 municipalities. Almost half of the regional area (51.2%) is devoted to agricultural use (SAT) for a total of approximately 1,220,000 h; of these, about 80% are actually used for agriculture (SAU). The most common crops are cereals (74% of SAU), followed by fodder, grassland, and woody crops (24% of SAU), and vineyards (2% of SAU).

### 3.2. Plant Protection Products: Sale Data and Toxicity

Table 2 provides data on the number (N) and total quantity (kg) of PPPs and active substances sold in Lombardy in 2016. A total of 2354 different products were sold for a quantity of about 1.3 × 10^7^ kg, including traditional non-organic products (85%) and organic products (15%). The number of active substances were 410 for a total of about 4.5 × 10^6^ kg, of which 370 were used in the traditional farming and 40 were used in the organic farming. Among non-organic products, the most sold were herbicides, followed by insecticides and fungicides. Among organic products, fungicides were the most sold (85%), while no products belonged to the class of herbicides. PPPs with multiple action constituted 14% of non-organic and 5% of organic products. Products not belonging to the classes of herbicides, fungicides, insecticides, and multi-action products have been combined into the category “other”, which accounts for 1% and 3% of the non-organic and organic products, respectively.

The density of use of non-organic PPPs and active substances was estimated to be 11.7 kg/h and 3.5 kg/h, respectively. For organic PPPs and active substances, the density of use was estimated to be 2 kg/h and 1.1 kg/h, respectively.

Table 3 shows the 10 best-sold non-organic active substances in 2016 and their classification, according to the CLP regulation [15]. Out of them, six were herbicides, two had multiple action, and two were fungicides. Glyphosate, a herbicide, was the most sold, and, together with glyphosate-isopropyl ammonium, its salt, represented the 30% of the 10 best-sold active substances. Altogether, the 10 best-sold active substances made up about 2.2 × 10^6^ kg, which was 63% of the total weight of all the active substances used in traditional farming.

### 3.3. Plant Protection Products in the Groundwater and Health Risk Assessment

Table 4 summarises the results of groundwater monitoring carried out by ARPA in 2016 [10]. Samples were taken from 157 and 158 monitoring points in spring and in autumn, respectively. Thirty-five molecules were monitored, which correspond to 31 active substances plus two metabolites for atrazine, one metabolite for glyphosate, and one for terbuthylazine. In the different monitoring points, the number of monitored molecules varied from 1 to 31. In addition to plant protection products, other parameters were contextually monitored, including inorganic compounds (such as sulfates, nitrates, and metal ions) and other organic pollutants, as well as some physicochemical parameters (pH, water temperature) not considered in this study.

Referring to traditional farming, of the 370 active substances currently sold in Lombardy, only 17 were submitted to official controls in the groundwater. Among them, only glyphosate, metolachlor, and terbuthylazine are among the 10 best-sold active substances. Notably, some of the monitored molecules were associated with substances banned since several years but still searched for due to their long environmental persistence. Monitored molecules are mainly herbicides. Comparing the concentration of active substances in groundwater with the EQS_single_, exceedances were observed for 16 molecules, including 12 active substances and four metabolites.

**Figure 1:**
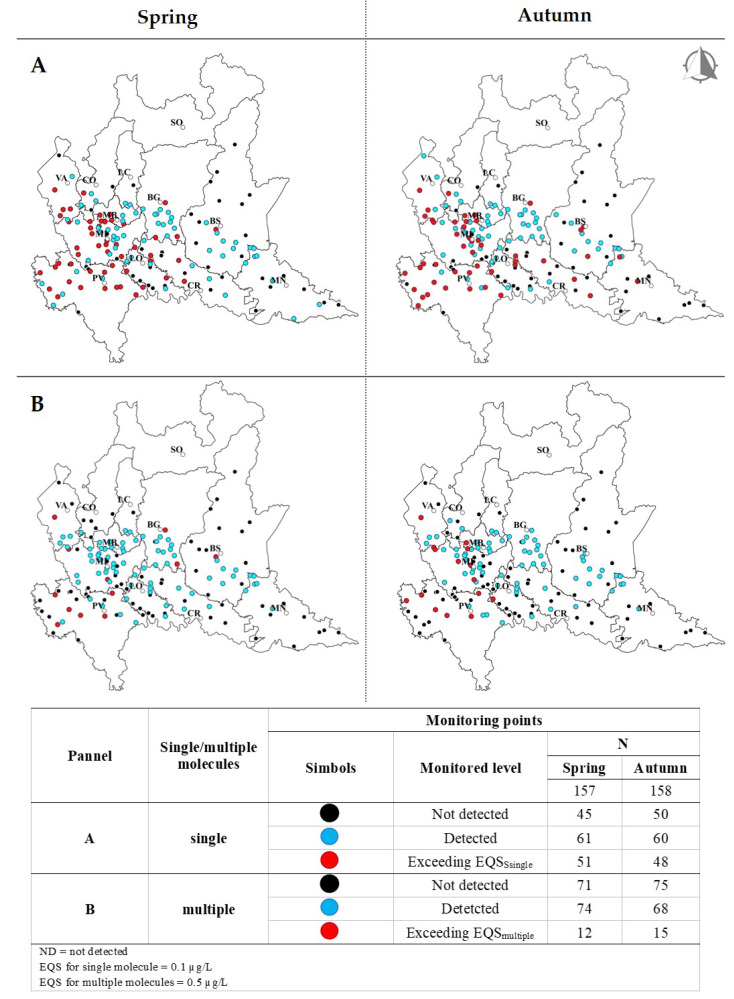
Maps of Lombardy, with the 12 provinces and their capitals (abbreviation). The monitoring points for groundwater are shown as dots. In panel (**A**,**B**): black dots = monitoring points where molecules were undetected. In panel (**A**): blue dots = points where at least one molecule was detected; red dots = points where one molecule exceeded the ESQ_single_. In panel (**B**): blue dots = points where two or more molecules were detected; red dots = points where two or more molecules exceeded the ESQ_multiple_. Results for the spring and the autumn campaign are shown.

Figure 1 shows the map of Lombardy, made with QGIS [24], with its 12 provinces. In panel A, the monitoring points for pesticides in the groundwater are reported as black, blue, and red dots, according with the absence of detection of any monitored molecule (black), the detection of at least one monitored molecule (blue), and the exceedance of the of EQS_single_ of at least one monitored molecule (red). The exceedances were found in 51 and 48 monitoring points (red dots), in spring and in autumn, respectively, for a total of 66 and 62 exceedances. Considering the total number of measurements carried out, that was 4532 in spring and 4620 in autumn, the exceedances were about 1.4% of measurements in both seasons. In about 39% of monitoring points in both seasons (61 in spring and 60 in autumn), molecules were detected, but with levels below the EQS_single_ (blue dots). In particular, in the province of Pavia (PV), the totality of monitoring points showed molecules exceeding the EQS_single_ in both campaigns. Among the best-sold molecules, glyphosate, metolachlor, and terbuthylazine had a number of exceedances ranging from 1 to 6, with a similar behavior in the two seasons; they were found in 1 to 4% of the monitoring points. In panel B, there are the monitoring points with multiple molecules detected or exceeding the EQS_multiple_. The exceedances were found in 12 and 15 monitoring points (red dots) in spring and autumn, respectively. In about 45% of monitoring points (74 in spring and 68 in autumn), multiple molecules were detected, but with levels below the EQS_multiple_ (blue dots).

Table 5 shows active substances with at least one exceedance of the EQS_single_, the number of exceedances, and the mean and maximum concentrations (see also red dots in Figure 1A). The active substance with the highest number of exceedances was bentazone, which is a herbicide used in rice crops, presenting 31 exceedances, with mean and maximum concentrations of 76 and 1319 µg/L, respectively; these concentrations were the highest among those detected in the groundwater. Other molecules with a frequency of exceedance >10 were atrazine, atrazine desethyl, dichlorobenil, and terbuthylazine desethyl, with a concentration below or equal to 1.1 µg/L. Considering the currently used pesticides, namely glyphosate, metolachlor, and terbuthylazine, the maximum concentration was up 3.4 µg/L, which was measured for glyphosate. Moreover, Table 5 reports the HQ for molecules exceeding the EQS_single_. HQ_mean_ ranged from 2.2 × 10^−4^ for bromacil to 1.2 × 10^−1^ for bentazone; the HQ_mean_ for the best-sold pesticides ranged from 2.4 × 10^−4^ for AMPA to 7.1 × 10^−3^ for terbuthylazine. The HQ_max_ ranged from 3.3 × 10^−4^ for bromacil to 2 for bentazone; for the best-sold pesticides, the HQ_max_ ranged from 4.9 × 10^−4^ for AMPA to 1 × 10^−2^ for terbuthylazine.

Table 6 shows results of the risk assessment for the monitoring points where two or more active substances and/or their metabolites were exceeding the EQS_multiple_ (red dots in Figure 1B). The risk was assessed separately for molecules grouped according to their systemic or local toxicity. For each monitoring point, the name and concentration of the molecules are reported, together with the HQ for each molecule and the HI for their sum. Referring to systemic toxicity, one monitoring point with exceedance of the EQS_multiple_ was found in spring; the active substances that contributed to the exceedance were *β*-hexachlorocyclohexane (not currently sold), atrazine, and terbuthylazine. Referring to local toxicity, exceedances of the EQS_multiple_ were found in seven monitoring points, of which three were in spring and four were in autumn; the active substances that contributed to the exceedances were bentazone, metolachlor, quinclorac, and molinate, which are all currently used pesticides except for molinate. The HI is 1.6 × 10^−2^ for the systemic toxicity, and it ranged from 1.1 × 10^−3^ to 8.1 × 10^−3^ for the local toxicity. The province of Pavia was the one with the largest number of monitoring points with multiple exceedances.

## 4. Discussion

In 2016, 2354 PPPs including 410 active substances were sold in Lombardy. Out of these, only 17 active substances, three of which belonged to the 10 most sold, were submitted to official controls for their presence in the groundwater. The risk for health associated with the intake to contaminated water in the adult general population was assessed.

The total amount of PPPs sold in Lombardy was 1.3 × 10^7^ kg (Table 2), accounting for about 9% of the total amount sold in Italy [1]. Indeed, the agricultural area used in Lombardy corresponds to 7.6% of the Italian agricultural area (12,885,186 h) [19], and the higher use of PPPs is probably associated with the intensive farming typical of the Po Valley.

The proportion between traditional and organic PPPs showed that 76% of products were traditional (Table 2). In fact, the Po Valley, covering the 75% of the agricultural area in Lombardy, is extensively cultivated with cereals. These crops are treated with different traditional PPPs, and particularly with herbicides, among which glyphosate is the best-sold active substance (Table 3). In the European Union, glyphosate is approved for its use as a herbicide and, following the harmonised classification [15], it is recognised as a substance with local toxicity that is able to cause serious damage to the eyes and environmental toxicity to aquatic life with long-term effects [25]. Glyphosate is also commercialised as isopropyl ammonium salt; this salt is also among the 10 best-sold traditional farming active substances (Table 3); according to the classification provided to ECHA for the registration process, isopropyl ammonium glyphosate is not classified as hazardous to humans [25]. Together, they accounted for the 21% of the traditional active substances sold in Lombardy in 2016. The other eight best-sold active substances were metam sodium, metazaclor, terbuthylazine, metam potassium, metolachlor, metiram, pendimetalin, and fosetil aluminium. According to their classification, their toxicity for humans is mostly due to local effects, with the notable exception of terbuthylazine and metazaclor, which may exert systemic effects, as terbuthylazine may cause damage to organs through prolonged or repeated exposure, and metazaclor is suspected of causing cancer.

In Italy, groundwater provides more than 70% of the national drinking water needed [26], and its quality must be guaranteed by national and local authorities. In 2016, the official controls on groundwater of ARPA Lombardia [10] concerned 31 traditional farming active substances, of which 25 were herbicides and six were persistent insecticides (aldrin, diedrin, edrin, isodrin, DDT, and lindane) banned years ago. This focus on herbicides may be due to their direct application on the ground and therefore the higher probability of leaching and contaminating the aquifer. However, only 17 out of the 370 traditional farming active substances were submitted to official controls (Table 4). In particular, we note that only four of the 10 best-sold traditional farming active substances were monitored; among those not monitored, there is the herbicide metazachlor, which is suspected of causing cancer. Moreover, in Italy, there is a lack of harmonisation in regional monitoring programs; the monitored molecules vary among regions; indeed, in 2016, on the whole Italian territory, 398 active substances were monitored and 259 were detected [27]. To this end, in 2018, a new national guideline for the design of the monitoring of PPPs in water, sediments, and biota was published [13]. This guideline recommends the adaptation of regional monitoring programs to substances actually placed on the market. In this regard, it is noteworthy that glyphosate, the best-sold active substance in Italy, was submitted to official controls in groundwater only in five Italian regions [27].

Of the 31 traditional farming active substances controlled in groundwater, 14 were not sold in 2016; these included some environmental persistent contaminants such as DDT, which is an insecticide banned in Italy in 1978. Other banned molecules submitted to official controls were atrazine, banned in 2008, aldrin, diedrin, edrin, isodrin, lindane, molinate, and simazine.

Of the large number of measurements carried out, over 9000, about 10% showed detectable concentrations of active substance, and less than 1.5% showed concentrations above the EQS_single_. These exceedances concern 11 active substances, of which four—atrazine, lindane, molinate and simazine—are persistent pollutants currently banned, and two, bromacil and dichlorobenil, were not sold in 2016 (Table 5). They contributed to the overall number of exceedances, with 67 vs. 61 exceedances for the five active substances currently used.

The presence of these contaminants in groundwater was recently reported in several studies. In particular, atrazine and its metabolite atrazine-desethyl were found in the United States, Republic of China, and France [28,29,30]; *β*-hexachlorocyclohexane (lindane) was reported in the Republic of China and Morocco [29,31]; dieldrin was reported in Italy [32]; and simazine was found in France, Morocco, and Italy [30,31,32]. Among these, for atrazine, *β*-hexachlorocyclohexane, dieldrin, and simazine, exceedances of the standards were observed, with maximum concentrations ranging from 0.210 to 221 µg/L [28,29,30,32,33].

Among the active substances currently in use, bentazone showed 31 exceedances of the EQS_single_, and mean and maximum concentrations of 76 and 1319 µg/L, respectively. Moreover, bentazone contributed largely to the overall number of exceedances of the EQS_multiple_, with the province of Pavia showing the largest number of monitoring points with exceedences. Bentazone is a herbicide used for the cultivation of rice, which is an important cereal in this province, where on an area of 85,000 h, equal to 1/10 of the regional utilised agricultural area, a total amount of 4.9 × 10^8^ kg of rice is produced each year, which represents the largest production at the European level [34]. The presence of bentazone in the groundwater is certainly caused by the mode of cultivation of the rice, which is grown in paddy water. Critical levels of bentazone were previously reported in groundwater samples [26,30,32,33], specifically related to Italian rice crops [26]. However, the toxicity of bentazone is mainly associated with local effects (allergic skin reaction and eye irritation, H317 and H319) and with acute toxicity (harmful is swallowed, H302), and it is not associated with long-term effects on humans [21].

Among the best-sold active substances, only 11 exceedances were found for glyphosate and its metabolite AMPA, while there were 13 exceedances for terbuthylazine and its metabolite terbuthylazine desetil and four exceedances for metolachlor. Together, they accounted for 0.3% of all performed measurements. For these molecules, the concentrations were always low, with a maximum value of 5.3 µg/L for AMPA. Previous studies reported exceedances for metolachlor, with maximum concentrations ranging from 0.2 to 12.5 µg/L [28,30,32,33] and for terbuthylazine, with maximum concentrations ranging from 0.7 to 29 µg/L [30,32,33]. Concentrations of glyphosate similar to those found in the present study were reported in Mexico, with maximum levels from 1.58 to 4.36 µg/L and a frequency of detection of 90–100% [35,36].

For molecules exceeding the EQS_single_, the HQ for the adult general population drinking contaminated water is typically in the order of 10^−3^–10^−4^ for all active substances, including the best-selling active ingredients glyphosate, terbuthylazine, and metolachlor (Table 5). Therefore, this is associated with a low risk for human health. The highest HQ was found for bentazone, with a mean value of 1.2 × 10^−1^ and a maximum of 2; however, the concentrations were associated with a HQ > 1 for only two monitoring points. To the best of our knowledge, this is the first study in Italy reporting data on pesticide human health risk assessment associated with the intake of drinkable water in the adult population. Actually, studies on pesticide human health risk assessment are scarce worldwide, and the majority of them regard agricultural area and/or specific compounds [29,31,37,38]. In a study conducted in an agricultural area of Morocco, groundwater contamination with pesticides and relative human health risk was evaluated by Berni et al.; the HQs of pesticides most frequently detected ranged from 2.3 × 10^−9^ for linuron to 1 × 10^−3^ for parathion ethyl [31]. Huang et al. reported the values of cumulative risk evaluated in groundwater samples collected from an important commodity grain base in China: four pesticides were detected in all samples, but their concentration did not pose a risk to adults except for two points where the risk quotient was close to 1 [29]. Li et al. evaluated the risk for atrazine, acetolachlor, hexachlorobenzene, and γ-HCH for 13 points from rural areas of China and reported risk values ranging from 4.6 × 10^−7^ for hexachlorobenzene to 8.6 × 10^−5^ for acetolachlor [37]. Finally, in an agricultural catchment dominated by cocoa crops in Ghana, Affum et al. evaluated the risk for both banned and current-use pesticides (five and nine, respectively) and reported values of HQ lower than 1 in all cases [38]. Recently, pesticides and pesticide degradates (more than 100 chemicals) were investigated in groundwater from public-supply wells across the US. Although these compounds occurred frequently, concentrations were low, and the risk for human health, both considering single pesticides and their mixture, was rarely approaching level of potential concern [28]. All these considering, our findings are in line with those of previous studies.

When the presence of multiple molecules in the groundwater was considered, it was found that in more than 44% of monitoring points, two to seven molecules were detected, of which about 9% exceeded the EQS_multiple_. The number of monitoring points with exceedances was similar in spring and autumn. Since the use of pesticides is much more abundant in spring than in autumn, this result may indicate that the exceedances were not associated with a recent use but rather with a previous pollution. This is confirmed by data of atrazine and its metabolites, the chemical with 28 exceedances, even though it was banned several years ago. Finally, for the assessment of the risk from exposure to multiple active substances, the method recently proposed by Goumenou and Tsatsakis [23] was adopted. Grouping the molecules according to their local or systemic health effects, we found a total of one exceedance of the EQS_multiple_ for molecules with systemic toxicity and seven exceedances for molecules with local toxicity. These exceedances were associated to a cumulative risk estimated as HI, which was 10^−2^ at the highest. These results testify a low risk for exposure to multiple pesticide residues with a common health effect following the ingestion of contaminated drinking water in the general adult population.

## 5. Conclusions

Overall, in Lombardy, Italy, more than 2300 PPPs containing more than 400 active substances are annually sold. Despite the large number and high amount of PPPs, the over 9000 measurements performed by official controls of groundwater revealed few exceedances (less than 1.5%) of the environmental quality standards and low concentrations. Based on these results, for the adult general population, the exposure to single and multiple active molecules from PPPs through drinking water was associated with an irrelevant risk for health, typically in the order of 10^−2^–10^−4^. However, the majority of the sold active substances, including the best-sold pesticides, was not included in the official controls, and this calls for an update of the list of active substances to be submitted to official controls, allowing a complete assessment of the risk for health.

## Figures and Tables

**Table 1:** Characteristics of the Lombardy territory and main agricultural crops.

Characteristics of the Territory	Unit	Parameter Value
Provinces	N	12
Municipalities	N	1539
Total area (ST)	h	2,382,132
Total agricultural area (SAT)		1,221,244
Utilised agricultural area (SAU)		980,112
SAT/ST	%	51.2
SAU/SAT		80.0
Cereals area/SAU		74
Vineyards area/SAU		2
Fruit and vegetables area/SAU		<1
Other crops area/SAU ^1^		24

^1^ Fodder, grassland and woody crops (excluding vineyards).

**Table 2:** Summary of the number (N) and quantity (kg) of PPPs sold in Lombardy in 2016, and active substances therein contained, divided into traditional farming and organic farming products/substances.

		Unit	Traditional Farming	Organic Farming
PPPs	Number	N	2354	
			2103	251
	Quantity	kg	1.3 × 10^7^	
	All classes	kg (%)	1.2 × 10^7^ (100)	2.0 × 10^6^ (100)
	Herbicides Fungicides Insecticides Multiple actions Other		5.6 × 10^6^ (49) 1.3 × 10^6^ (12) 2.8 × 10^6^ (24) 1.7 × 10^6^ (14) 9.8 × 10^4^ (1)	- 1.7 × 10^6^ (85) 1.4 × 10^5^ (7) 9.8 × 10^4^ (5) 6.2 × 10^4^ (3)
Active substances	Number	N	410	
			370	40
	Quantity	kg	4.5 × 10^6^	
			3.5 × 10^6^	1.1 × 10^6^

**Table 3:** Ten best-sold traditional farming active substances in Lombardy in 2016, their functional class, the sold quantity, and their hazard classification according to CLP EU regulation.

Active Substance	CAS	Functional Class	Quantity (kg)	Classification (CLP EU)	
				Health Hazards	Environmental Hazards
Glyphosate	1071-83-6	herbicide	6.0 × 10^5^	H318	H411
Metam Sodium	137-42-8	multiple action	4.8 × 10^5^	H302, H314, H317	H400, H410
Metazaclor	67129-08-2	herbicide	4.0 × 10^5^	H317, H351	H400, H410
Terbuthylazine	5915-41-3	herbicide	1.9 × 10^5^	H302, H373	H400, H410
Metam Potassium	137-41-7	multiple action	1.3 × 10^5^	H302, H312, H314, H317, H332	H400, H410
Metolachlor	51218-45-2	herbicide	1.3 × 10^5^	H317, H330	H400, H410
Metiram	9006-42-2	fungicide	7.9 × 10^4^	H317, H330	-
Pendimetalin	40478-42-1	herbicide	6.1 × 10^4^	H317	H400, H410
Glyphosate-isopropylammonium	38641-94-0	herbicide	5.2 × 10^4^	-	H411
Fosetil Aluminium	39148-24-8	fungicide	5.0 × 10^4^	H318	-
Total			2.2 × 10^6^		

**Table 4:** Summary of official controls of groundwater in Lombardy by ARPA in 2016. List of the active substances and of the molecules monitored, with the identification of those sold in the year 2016. Number of monitoring points and number and percentage of monitoring points in which at least one exceedance of the EQS_single_ was found, in the spring and autumn campaign.

Active Substance	Monitored Molecule	Active Substance Sold in 2016	Monitoring Points (n)		Monitoring Points with Exceedances n (%)	
			Spring	Autumn	Spring	Autumn
(MCPA) 2,4 acetic meticlorophenoxy acid	(MCPA) 2,4 acetic meticlorophenoxy acid	✓	134	131	0	0
2,4 dichlorophenoxy acetic acid (2,4 D)	2,4 dichlorophenoxy acetic acid (2,4 D)		107	95	0	0
Alachlor	Alachlor	✓	126	123	0	0
Aldrin	Aldrin		147	148	0	0
Atrazine	Atrazine		126	123	9 (7.1)	5 (4.1)
	Atrazine-desethyl		126	123	6 (4.8)	5 (4.1)
	Atrazine-desisopropyl		126	123	1 (0.8)	2 (1.6)
Azimsulfuron	Azimsulfuron	✓	107	105	0	0
Bensulfuron methyl	Bensulfuron methyl		107	105	0	0
Bentazone	Bentazone	✓	134	131	16 (11.9)	15 (11.5)
Lindane	*β*-Hexachlorocyclohexane		126	123	1 (0.8)	2 (1.6)
Bromacil	Bromacil		127	124	3 (2.4)	1 (0.8)
DDD, DDT, DDE	DDD, DDT, DDE		73	71	0	0
Dicamba	Dicamba	✓	134	131	0	0
Dichlorobenil	2,6-Dichlorobenzamide		127	124	11 (8.7)	13 (10.5)
Diedrin	Diedrin		147	148	0	0
Dimethoate	Dimethoate	✓	100	99	0	0
Diuron	Diuron	✓	108	124	0	0
Endrin	Endrin		147	-	0	0
Glyphosate	Glyphosate	✓	132	131	1 (0.8)	2 (1.5)
	AMPA		125	123	5 (4.0)	3 (2.4)
Imidacloprid	Imidacloprid	✓	107	105	0	0
Isodrin	Isodrin		147	-	0	0
Isoproturon	Isoproturon		115	131	0	0
Linuron	Linuron	✓	134	131	0	0
Mecoprop	Mecoprop	✓	134	131	0	0
Metolachlor	Metolachlor	✓	126	123	3 (2.4)	1 (0.8)
Molinate	Molinate		126	123	3 (2.4)	3 (2.4)
Nicosulfuron	Nicosulfuron	✓	107	105	0	0
Propanil	Propanil	✓	127	124	0	0
Quinclorac	Quinclorac	✓	107	105	0	2 (1.9)
Simazine	Simazine		126	123	1 (0.8)	1 (0.8)
Sulcotrione	Sulcotrione	✓	115	131	0	0
Terbuthylazine	Terbuthylazine	✓	126	123	2 (1.6)	1 (0.8)
	Terbuthylazine desethyl		125	122	4 (3.2)	6 (4.9)
31	35	17	157	158	51	48

**Table 5:** Active substances for which at least one exceedance of the EQS_single_ was observed, number of exceedances and measured concentrations, acceptable daily intake (ADI) and hazard quotient (HQ) for the health risk for an adult ingesting 2 L of drinking water per day, based on the mean and maximum concentrations.

Active Substance	Monitored Molecule	Hazard Identification (CLP)	No. of Exceedances	Concentration of Exceedances (µg/L)		20% ADI (mg/kg Body Weigth)	HQ	
				Mean	Maximum		HQ_mean_	HQ_max_
Atrazine	Atrazine	H317, H373	14	0.1	0.4	0.004	7.1 × 10^−4^	2.9 × 10^−3^
	Atrazine-desethyl	H302, H319, H332	11	0.1	0.3	0.004	7.1 × 10^−4^	2.1 × 10^−3^
	Atrazine-desisopropyl	H302, H319, H332	3	0.2	0.2	0.004	1.4 × 10^−3^	1.4 × 10^−3^
Bentazone	Bentazone	H302, H317, H319	31	76.3	1319	0.018	1.2 × 10^−1^	2.0
Bromacil	Bromacil	H302, H315, H319	4	0.2	0.3	0.026	2.2 × 10^−4^	3.3 × 10^−4^
Dichlorobenil	2,6-Dichlorobenzamide	H312	24	0.3	1.1	0.002	4.3 × 10^−3^	1.6 × 10^−2^
Glyphosate	Glyphosate	H318	3	1.8	3.4	0.2	8.1 × 10^−4^	2.5 × 10^−3^
	AMPA	H302, H314, H315, H319, H332	8	1.7	5.3	0.06	2.4 × 10^−4^	4.9 × 10^−4^
Lindane	*β*-Hexachlorocyclohexane	H301, H311, H312, H330, H351	3	0.4	0.5	0.001	1.1 × 10^−2^	1.4 × 10^−2^
Metolachlor	Metolachlor	H317, H330	4	0.9	3.0	0.02	1.3 × 10^−3^	4.3 × 10^−3^
Molinate	Molinate	H302, H317, H332, H351, H361f, H373	6	0.1	0.4	0.0016	1.8 × 10^−3^	7.1 × 10^−3^
Quinclorac	Quinclorac	H317	2	2.6	4.9	0.08	9.3 × 10^−4^	1.7 × 10^−3^
Simazine	Simazine	H351	2	0.1	0.1	0.004	7.1 × 10^−4^	7.1 × 10^−4^
Terbuthylazine	Terbuthylazine	H351	3	0.2	0.3	0.0008	7.1 × 10^−3^	1.0 × 10^−2^
	Terbuthylazine desethyl	H302, H373	10	0.1	0.2	0.0008	3.6 × 10^−3^	7.1 × 10^−3^

**Table 6:** Summary of monitoring points where two or more monitored molecules, grouped by systemic or local toxicity, exceeded the EQS_multiple_. For each molecule, the risk for health for an adult ingesting 2 L of drinking water per day was assessed as hazard quotient (HQ). For each groundwater sample the cumulative risk was assessed as hazard index (HI).

Season	Monitoring Point	Province	Monitored Molecules	Levels (µg/L)	HQ	HI
Systemic toxicity						
Spring	PO0170290UB135	BS	Atrazine	0.01	7.1 × 10^−5^	1.6 × 10^−2^
			*β*-hexachlorocyclohexane	0.54	1.5 × 10^−2^	
			Terbuthylazine	0.01	4.6 × 10^−4^	
Local toxicity						
Spring	PO0161010R0004	BG	Metolachlor	3.00	4.3 × 10^−3^	4.6 × 10^−3^
			Terbuthylazine desethyl	0.01	3.6 × 10^−4^	
	PO018003NR0009	PV	Bentazone	0.67	1.1 × 10^−3^	1.1 × 10^−3^
			Quinclorac	0.03	1.2 × 10^−5^	
	PO018162NUP001	PV	Bentazone	0.33	5.2 × 10^−4^	7.8 × 10^−3^
			Molinate	0.41	7.3 × 10^−3^	
Autumn	PO098012NR0011	LO	Bentazone	0.23	3.7 × 10^−4^	2.2 × 10^−3^
			Quinclorac	0.24	8.4 × 10^−5^	
			Terbuthylazine desethyl	0.05	1.8 × 10^−3^	
	PO018003NR0009	PV	Bentazone	1.48	2.3 × 10^−3^	2.4 × 10^−3^
			Quinclorac	0.08	2.8 × 10^−5^	
	PO018176NUP001	PV	Bentazone	1.06	1.7 × 10^−3^	3.5 × 10^−3^
			Molinate	0.10	1.8 × 10^−3^	
	PO018162NUP001	PV	Bentazone	0.27	4.2 × 10^−4^	8.1 × 10^−3^
			Molinate	0.43	7.7 × 10^−3^	

## Data Availability

Databases accessed for this research are ICPS—fitoWeb290—Regional register of sales of plant protection products. Available online: http://www.icps.it/fitoweb290 (last accessed on 18 March 2021); ARPA Lombardy—Environmental Protection Regional Agency. Data on the monitoring of quality of groundwater bodies (2016). Available online: https://www.arpalombardia.it/Pages/Dati/Valori-analitici-Sotterranee-2016.aspx?tipodati=0&tema=Acque&sottotema=Acque%20sotterranee&anno=2016&ordine=1 (last accessed on 18 March 2021); ASR Annuario Statistico della Regione Lombardia. Comuni, superficie territoriale, popolazione residente media e densità media al 31.12.—Italia, Lombardia e province lombarde (asr-lombardia.it). Available online: https://www.asr-lombardia.it/asrlomb/ (last accessed on 25 March 2021); IStat—Istituto Nazionale di Statistica—6° Censimento generale dell’agricoltura. Istat.it—6° Censimento agricoltura 2010. Available online: https://www4.istat.it/it/censimento-agricoltura/agricoltura-2010 (last accessed on 25 March 2021).

## Supplementary Materials

The following are available online at https://www.mdpi.com/article/10.3390/toxics9070160/s1. Table S1: Legend of Hazard Statements.
