# Supplementary material for: Use of Plant Protection Products in Lombardy, Italy and the Health Risk for the Ingestion of Contaminated Water
Source: Toxics. 2021 Jul 6;9(7):160. doi: 10.3390/toxics9070160 (PMC8309806; doi:10.3390/toxics9070160)
Supplement: Supplementary file 1 [file toxics-09-00160-s001.zip › toxics-1187711-supplementary.pdf]

Supplementary Materials: Use of Plant Protection Products in Lombardy, Italy and the Health Risk for the Ingestion of Contaminated Water

Rosa Mercadante, Beatrice Dezza, Teresa Mammone, Angelo Moretto and Silvia Fustinoni

Table S1. Legend of Hazard Statements.

Code	**Hazard Statements**
H301	Toxic if swallowed
H302	Harmful if swallowed
H303	May be harmful if swallowed
H311	Toxic in contact with skin
H312	Harmful in contact with skin
H314	Causes severe skin burns and eye damage
H315	Causes skin irritation
H317	May cause an allergic skin reaction
H318	Causes serious eye damage
H319	Causes serious eye irritation
H330	Fatal if inhaled
H332	Harmful if inhaled
H351	Suspected of causing cancer
H373	May cause damage to organs through prolonged or repeated exposure
